# Simulation of Magnetodielectric Effect in Magnetorheological Elastomers

**DOI:** 10.3390/ijms20061457

**Published:** 2019-03-22

**Authors:** Danil Isaev, Anna Semisalova, Yulia Alekhina, Liudmila Makarova, Nikolai Perov

**Affiliations:** 1 Faculty of Physics, Lomonosov Moscow State University, 119991 Moscow, Russia; semisalova@magn.ru (A.S.); ya.alekhina@physics.msu.ru (Y.A.); la.loginova@physics.msu.ru (L.M.); perov@magn.ru (N.P.); 2 Helmholtz-Zentrum Dresden-Rossendorf, Institute of Ion Beam Physics and Materials Research, 01328 Dresden, Germany; 3 Institute of Physics, Matematics & IT, Baltic Federal University, 236041 Kaliningrad, Russia

**Keywords:** magnetorheological elastomers, magnetoactive elastomers, magnetodielectric effect, numerical modeling

## Abstract

We present the results of numerical simulation of magnetodielectric effect (MDE) in magnetorheological elastomers (MRE)—the change of effective permittivity of elastomer placed under the external magnetic field. The computer model of effect is based on an assumption about the displacement of magnetic particles inside the elastic matrix under the external magnetic field and the formation of chain-like structures. Such displacement of metallic particles between the planes of capacitor leads to the change of capacity, which can be considered as a change of effective permittivity of elastomer caused by magnetic field (magnetodielectric effect). In the literature, mainly the 2D approach is used to model similar effects. In this paper, we present a new approach of magnetorheological elastomers simulation—a 3D-model of the magnetodielectric effect with ability to simulate systems of $10^{5}$ particles. Within the framework of the model, three types of particle size distributions were simulated, which gives an advantage over previously reported approaches. Lognormal size distribution was shown to give better qualitative match of the modeling and experimental results than monosized type. The developed model resulted in a good qualitative agreement with all experimental data obtained earlier for Fe-based elastomers. The proposed model is useful to study these novel functional materials, analyze the features of magnetodielectric effect and predict the optimal composition of magnetorheological elastomers for further profound experimental study.

## 1. Introduction

Due to the variety of their properties, magnetorheological materials belong to the class of so-called smart materials. They can respond to changes in their environment and actively alter some physical properties according to these changes. Such responsiveness defines the practical importance of these materials and their wide use in different fields from everyday exploitation to industrial applications [1,2,3,4,5,6].

Magnetorheological elastomers (MRE) usually consist of a soft polymer matrix with magnetic particles dispersed in it [7,8,9,10,11,12]. Recent experimental studies show that MRE exhibit mechanical and electrical property changes under the external magnetic field: magnetorheological effect [13], magnetoresistance effect [14], magnetodielectric effect (MDE) [15], etc. The property changes of MRE may be explained by the displacement of particles inside the matrix. Particles tend to concentrate to column-like structures along the direction of the external magnetic field. This leads to the shape deformation of the sample and causes the anisotropy of effects.

Bica et al. studied the variation of capacitance of capacitor filled with MRE, which was dependent on the distance between the plates [16,17]. They also studied the concentration dependence of MDE [18], its anisotropy [19], and electroconductive properties [20]. Yu et al. studied wave-absorbing properties of MRE [21] and conductivity of magnetorheological gels [22]. Knicht et al. introduced the model of contact resistance and studied piezoresistivity of MRE [23]. In [24], they extended their model for thermoresistance and showed the decrease of resistance of MRE with the external field increase. Wang et al. studied the fatigue dependences of properties of MRE [25]. They noted that capacitance tends to increase for the same value of magnetic field for “older” materials because of breaking of their inner structures. Semisalova et al. [15] reported the significant change of effective permittivity of MRE measured using plane capacitor with fixed distance between the plates. Kramarenko et al. studied hysteresis characteristics of MDE in magnetoactive materials [26]. In [27], Tsai et al. studied influence of conductive particles chains on permittivity of elastomer. They showed that organizing particles into chain-like structures may increase permittivity of material by 85% compared to random distribution of filler particles.

There are several different approaches to the theoretical estimation of such effects. Homogenization and estimation through so-called effective medium may be used for mechanical properties. Electrical properties may be estimated through an influence of the single regular chain of particles or the imagine filler as cuboids with changing linear dimensions.

The goal of this work is to give good qualitative description of magnetodielectric effect—the change of capacity of a plane capacitor filled with magnetic elastomer and placed under the external magnetic field. In this work, we present a model and results of the direct numerical modeling of the magnetodielectric effect in MRE.

## 2. Model

**Figure 1:**
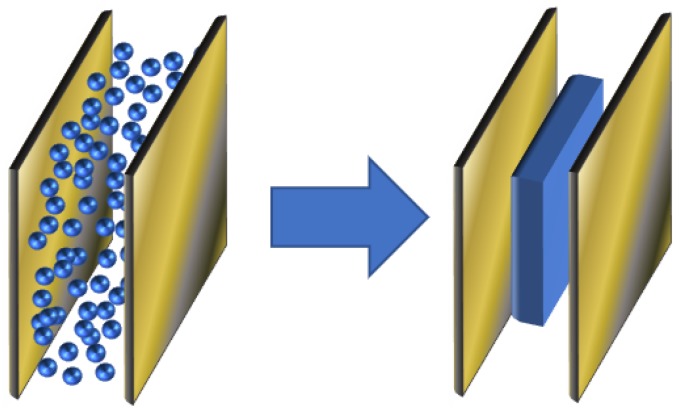
Plane capacitor with equivalent metal cuboid placed inside [15].

In [15], a simple qualitative model of MDE is presented. It is based on representation of magnetic filler as metal cuboid, which gives simple formula for capacitance evaluation. When an external magnetic field is applied, linear dimensions are changed correspondingly to reflect the change of internal particles structure (see Figure 1). In this work, we extend this model to be more accurate, yet still be simple in terms of numerical calculations. We use a similar approach to estimate capacitance, but take into account actual particles distribution within MRE between the capacitor planes.

To do that, we consider MRE as a system of spherical particles in the elastic matrix. This system is affected by the homogeneous magnetic field. Each particle in such system is in the field of magnetic dipoles, the external magnetic field and in the elastic forces field. We assume that filler particles are the soft magnetic material and the external magnetic field can only orient magnetic moments of particles. Therefore, the moments of particles are oriented towards effective field, which is the sum of the external field and nearest neighbors’ gradient field. Particles move under influence of the gradient field generated by the nearest neighbors, and the field of elastic forces generated by the particles’ displacement in polymer matrix relative to their initial equilibrium position.

### 2.1. Magnetic Force

We calculate gradient magnetic field on given particle as sum of dipole field of particle nearest neighbors ${\vec{H}}_{nn}$:

(1) $${\vec{H}}_{nn}=\sum_{k=1}^{N}{\vec{H}}_{k}=\sum_{k=1}^{N}\frac{3\left({\vec{m}}^{k}{\vec{R}}_{k}\right){\vec{R}}_{k}-R_{k}^{2}{\vec{m}}^{k}}{R_{k}^{5}},$$

where ${\vec{m}}^{k}$ is the magnetic moment of *k*th neighbor, and $R_{k}$ is the distance between the given particle and neighbor particle.

Thus, for magnetic force $F^{magn}$ acting on the particle, we obtain the following equation:

(2) $${\vec{F}}^{magn}=\nabla {\vec{H}}_{nn}\vec{m}$$

Consequently, for each projection:

(3) $$\begin{array}{c} F_{x_{j}}^{magn}=\sum_{x_{i}}m_{x_{i}}\frac{dH_{x_{i}}}{dx_{j}} \end{array}$$

(4) $$\begin{array}{c} \frac{dH_{x_{i}}}{dx_{j}}=\sum_{k=1}^{N}\frac{A_{k}R_{k}^{2}-5B_{k}\left(x_{j}-x_{j}^{k}\right)}{R_{k}^{7}}, \end{array}$$

(5) $$\begin{array}{c} A_{k}=3m_{x_{j}}^{k}\left(x_{i}-x_{i}^{k}\right)+3\left({\vec{m}}^{k}{\vec{R}}_{k}\right)\delta_{ij}-2\left(x_{j}-x_{j}^{k}\right)m_{x_{i}}^{k}, \end{array}$$

(6) $$\begin{array}{c} B_{k}=3\left({\vec{m}}^{k}{\vec{R}}_{k}\right)\left(x_{i}-x_{i}^{k}\right)-R_{k}^{2}m_{x_{i}}^{k} \end{array}$$

where $x_{i}$ and $x_{j}$ are independent coordinate axes (x,y,z), $\delta_{ij}$ is Kronecker delta, $\vec{m}$ is the magnetic moment of the particle, and *x* and $x^{k}$ are coordinates of a particle and its *k*th neighbor.

### 2.2. Elastic Force

**Figure 2:**
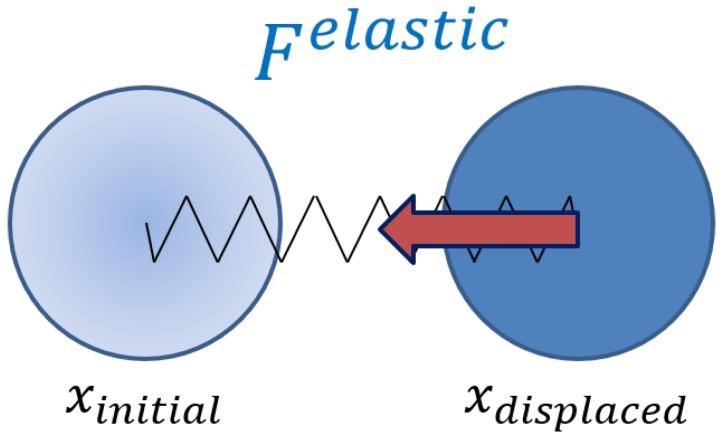
When a particle is moved from its initial position $x_{initial}$, the elastic returning force $F^{elastic}$ appears.

For elastic interaction of particles with the polymer matrix, we use simple spring approach [28,29]. Each particle is assumed to be connected with its initial position by a “virtual” spring (see Figure 2). When a particle is moved from its initial position, the spring stretches and acts to return particle back. Thus, each particle is assumed to be in the field of elastic force, which tends to return the particle to the initial position if a displacement happens.

We obtain this force from the elastic modulus of MRE *E* defined as follows:

(7) $$E=\frac{F^{elastic}/S}{\delta l/l}=\frac{F^{elastic}l}{S\delta l},$$

where *S* is an area, *l* is the length of deforming media and δl is the displacement.

We assume $S=\pi r^{2}$ to be an area of a diametral cross-section of the spherical particle, and *l* to be average distance between particles. For the elastic force $F^{elastic}$ acting on the particle, we obtain:

(8) $$F^{elastic}=\pi E\frac{r^{2}}{l}\delta l$$

### 2.3. Numerical Method

Each particle is located in the dipole field of its neighbors and in the field of elastic forces, thus the equation of particle motion is:

(9) $$\begin{array}{c} m\frac{d^{2}\vec{r}\left(t\right)}{dt^{2}}={\vec{F}}^{magn}+{\vec{F}}^{elastic}\\ \vec{r}\left(0\right)={\vec{r}}_{0}\\ \frac{d\vec{r}\left(0\right)}{dt}=0. \end{array}$$

For each particle, we set the initial position, and set the velocity of the particles to zero. To solve Equation (9) numerically, we used the Verlet algorithm [30].

In short, the difference scheme may be obtained in the following way. We approximate the function r(t) in *h*-neighborhood of *t* as Taylor series:

(10) $$\begin{array}{c} r\left(t+h\right)=r\left(t\right)+\frac{d\;r}{d\;t}h+\frac{1}{2}\frac{d^{2}r}{dt^{2}}h^{2}+\frac{1}{6}\frac{d^{3}r}{dt^{3}}h^{3}+O\left(h^{4}\right),\\ r\left(t-h\right)=r\left(t\right)-\frac{d\;r}{d\;t}h+\frac{1}{2}\frac{d^{2}r}{dt^{2}}h^{2}-\frac{1}{6}\frac{d^{3}r}{dt^{3}}h^{3}+O\left(h^{4}\right). \end{array}$$

After summation and substitution, we get:

(11) $$r\left(t+h\right)=2r\left(t\right)-r\left(t-h\right)+\frac{1}{m}F\left(t\right)h^{2}+O\left(h^{4}\right).$$

Consequently, assuming $h=t_{i+1}-t_{i}$:

(12) $$r_{i+1}=2r_{i}-r_{i-1}+\frac{1}{m}F_{i}h^{2}+O\left(h^{4}\right).$$

Thus, the position of each particle at each iteration depends on two previous positions and does not depend on particle speed. This equation represents the Verlet integration.

### 2.4. Capacitance Calculation

An important part of our model is the calculation of capacitance. In [15], capacitance *C* is estimated through the equivalent system when all particles are replaced with a metallic cuboid with the volume equal to the total volume of particles. In this work, we aim to improve accuracy of this method, but preserve its simplicity based on the area cap approximation:

(13) $$C=\epsilon_{0}\epsilon \frac{S}{d},$$

where *S* and *d* are corrected for lateral sizes of metallic inclusion between the planes of capacitor. This allows us to estimate capacitance change caused by particles displacement relative to each other.

**Figure 3:**
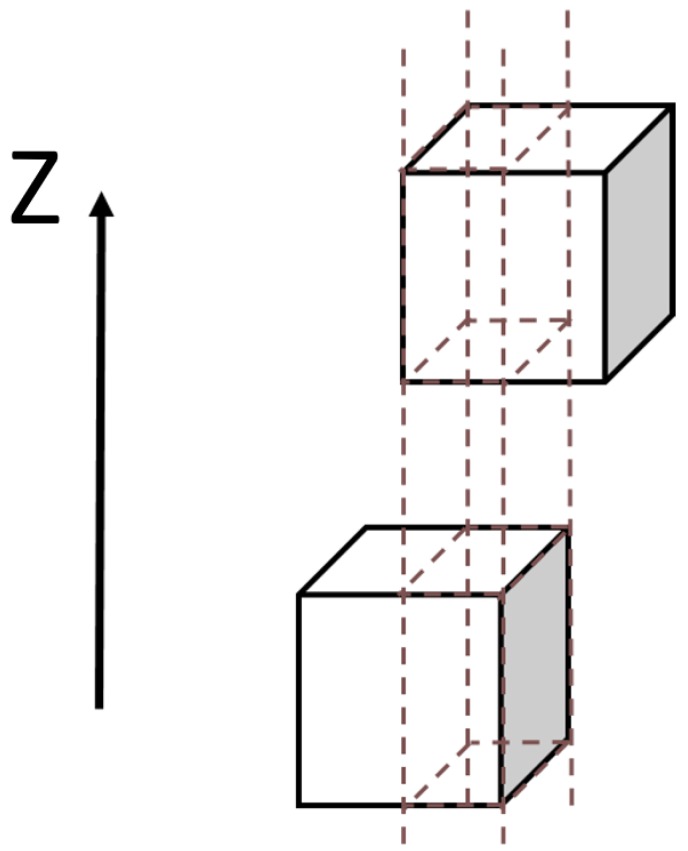
Split the volume along *z*-axis.

To use area cap approach, we approximate spherical particles with axis-aligned shapes (cuboids). In addition, we assume that electric field inside the capacitor is not changed and remains uniform and constant. Therefore, we can approximate our sample as a system of interconnected planar capacitors. To determine a total capacitance, we split volume along the z-axis, as shown on Figure 3, which is perpendicular to the capacitors plates, into areas, so that each particle either completely overlaps this area or does not overlap at all. In this way, each slice along z-axis is a series of capacitors, while the slices are connected in parallel. To obtain such partition, we need to review projections of particles onto the plane XY. Therefore, the problem is reduced to the analysis of two-dimensional geometry.

**Figure 4:**
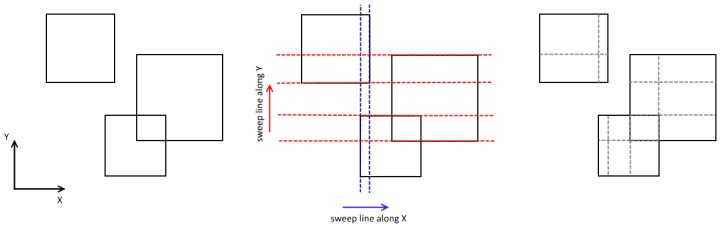
Sweep line example: (**Left**) initial state (projections of particles on XY plane); (**Middle**) sweep line cut area; and (**Right**) final split.

To solve this problem, the sweep line algorithm was used. The sweep line is an imaginary line running along the *x*-axis in one direction. It processes “events”—in our case an event is the crossing with particle boundaries. After registration of two consecutive events, we get the narrow band at which second line starts running along the *y*-axis, splitting the particles projection at each “event” of crossing the particle borders (Figure 4).

### 2.5. Parameters

We implemented the algorithm using C++ programming language to solve Equation (9). For all simulations, a standard personal computer was used. We chose Fe as particles’ material and used sample parameters similar to in the experiment (see Section 4). Particles had radius $R_{0}$ of 5 μm. Young’s modulus $E_{0}$ of elastomer matrix was varied from 0.01 GPa to 1 GPa. We did not use any periodical approximations and simulated sample as whole using 25,000–130,000 particles in each simulation. Volume concentration of metallic particles was varied up to 27%.

One of advantages of this model is its simplicity in terms of calculating. This allowed us to solve Equation (9) for a system of $10^{5}$ particles with capacitance evaluation (average modeling point) in less than 1 min on an ordinary laptop.

### 2.6. Particles Size Distributions

**Figure 5:**
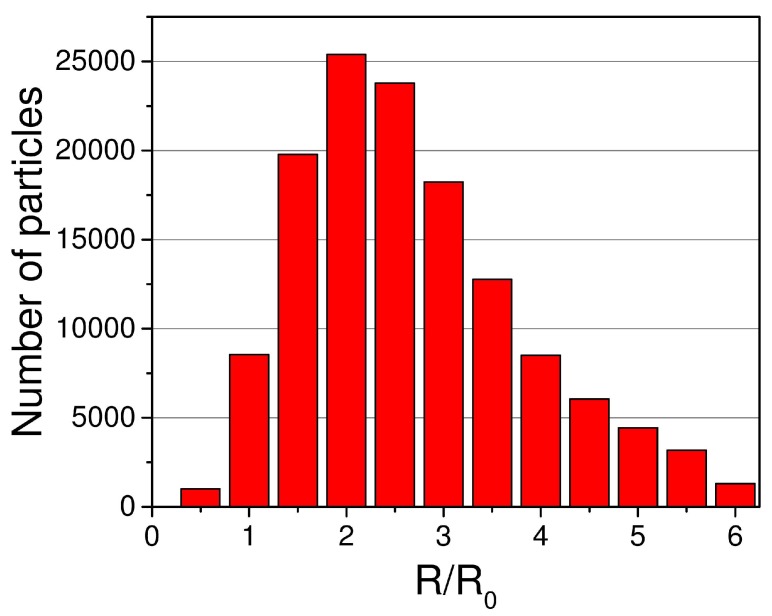
Lognormal distribution of 130,000 particles with median 1 and sigma 0.7. $R_{0}$ corresponds to radius of 5 μm.

Capacitance of systems of many particles strongly depends on particles mutual positions and sizes. The MDE value may be dependent on the type of particles size distribution. To investigate this, we considered three types of particle size distributions. Monosized with all particles of the same size was used as a base type. Lognormal type was obtained by modulating monosized radius with lognormal distribution with median 1 and sigma 0.7 (Figure 5). We also used bimodal as intermediate type with two types of particles size, namely small and large, with three times volume differences between each other.

## 3. Results and Discussion

The aim of this work was to check the model qualitative behavior and compare it with experimental data. Thus, data are presented in normalized units, i.e., divided by maximum value.

### 3.1. Magnetic Properties

**Figure 6:**
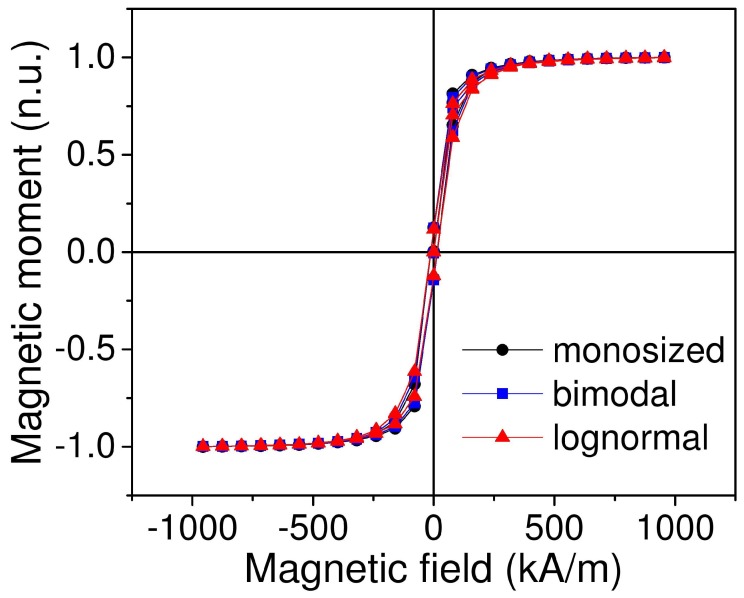
Simulated field dependence of magnetization of magnetorheological elastomers (MRE) with different types of size distribution of filler particles. Simulation was performed for a sample with 8.1% volume concentration of Fe.

**Figure 7:**
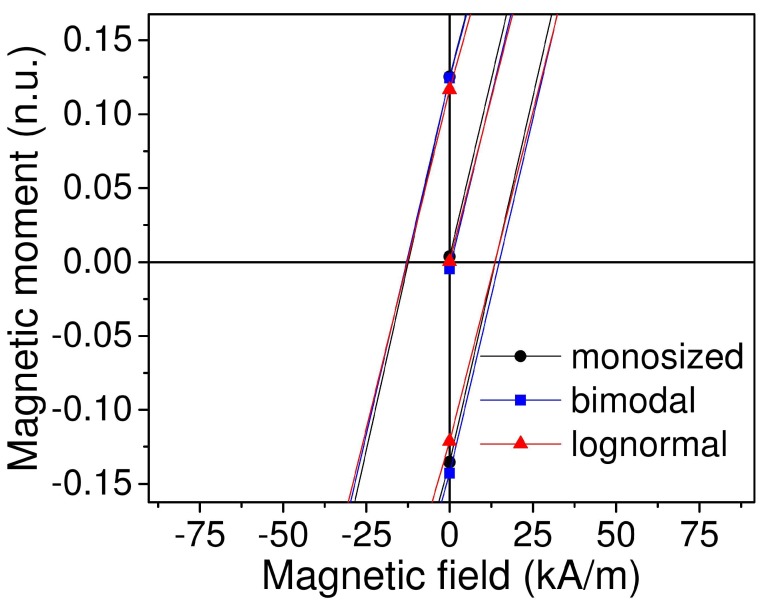
Low-field region showing the open hysteresis loop.

**Figure 8:**
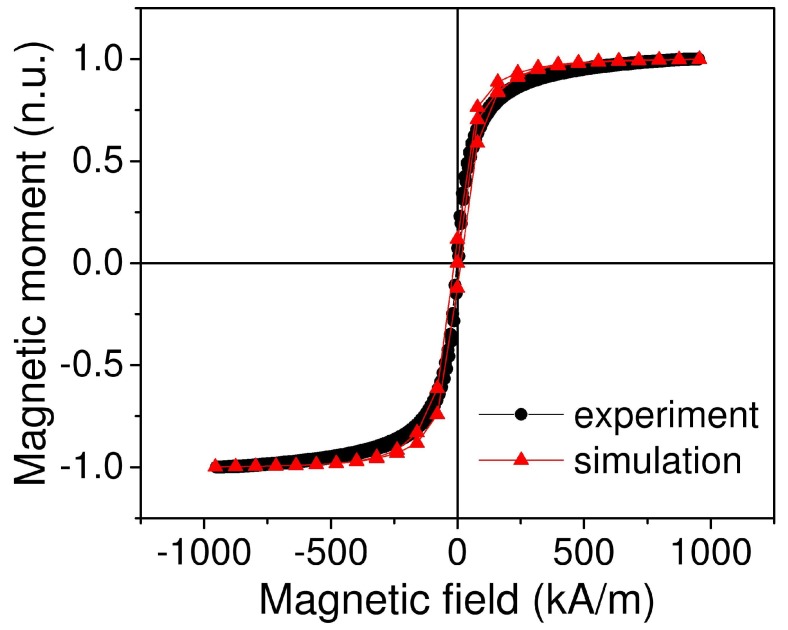
The comparison of experimental and simulated magnetization of MRE vs. applied magnetic field. Experiment was performed for the MRE sample with 8.1% volume concentration of Fe. Simulation was performed for the sample with 8.1% volume concentration of Fe, lognormal size distribution.

First, we simulated magnetic properties of the material, particularly the magnetic field dependence of magnetization. Simulation results show that we obtained the same magnetic hysteresis for different types of particle size distribution—monosized, bimodal and lognormal (Figure 6). In addition, the simulation showed the presence of coercive field for all three types of MRE (Figure 7). The simulated field dependence matcheed well with the experimental results. Figure 8 shows the comparison of magnetic hysteresis curves obtained using our model and measured with vibrating sample magnetometer (VSM). For magnetization measurements with VSM, sample was attached to the holder with Teflon tape. We assumed that a minor change of sample shape would not affect demagnetization factor significantly and thus neglected it.

### 3.2. Magnetodielectric Effect

**Figure 9:**
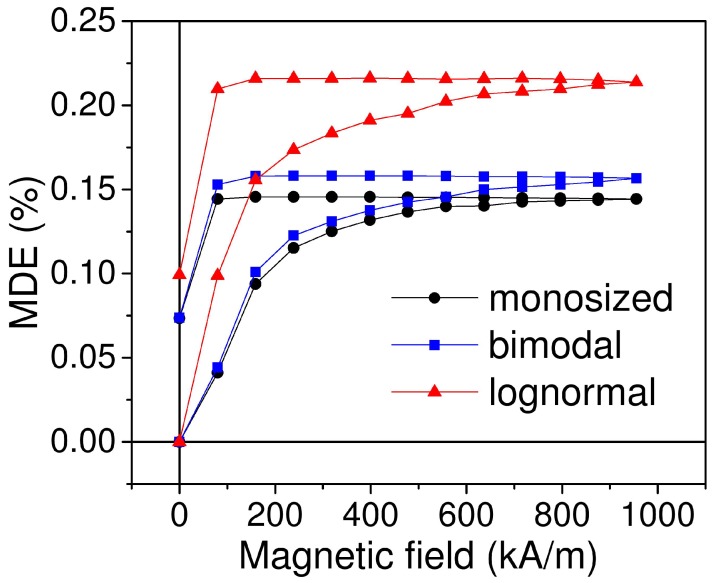
Simulated field dependence of magnetodielectric effect (MDE) for three different types of size distribution. Volume concentration of magnetic particles is 25%; magnetic field is perpendicular to the capacitor plane.

The simulated field dependences of MDE in magnetic elastomers with three types of particles size distribution are shown in Figure 9. All three types of samples show the same qualitative behavior, hence they are different in the maximum MDE value, with lognormal distribution being the largest one.

**Figure 10:**
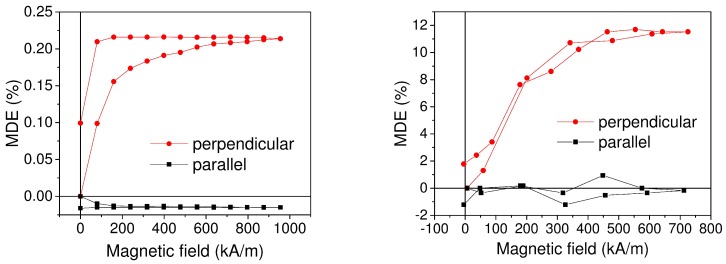
Comparison of simulated field dependence of MDE (**left**) with experimental data (**right**) for the magnetic field applied perpendicular and parallel to the capacitor plane. Simulation: Sample with 25% volume concentration of Fe, lognormal size distribution. Experiment: Sample with 25.3% volume concentration of Fe. Model showed good qualitative agreement.

We compared simulation results for dependence of MDE on external magnetic field with experimental data reported in [15] and obtained for Fe-based MRE (Section 4). Comparison showed that the developed model described the effect qualitatively well (Figure 10).

Significant quantitative discrepancy may be partly explained with difference in parameters of studied samples. Experimental data were obtained for samples with elastic modulus 100 times smaller than we used in modeling, and we have shown (see Section 3.4) the significant change of the effect with elastic modulus variation. The presented approach of using “virtual springs” for particle–matrix interaction was limited in the application to less rigid polymer samples, which were studied experimentally.

The anisotropy of MDE was confirmed within our model. We observed the difference in the effect sign and magnitude for different directions of the external field. In addition, the hysteretic character of field dependence due to the polymer matrix elasticity was found similar to experimental observations.

### 3.3. Concentration Dependence of Magnetodielectric Effect (MDE)

**Figure 11:**
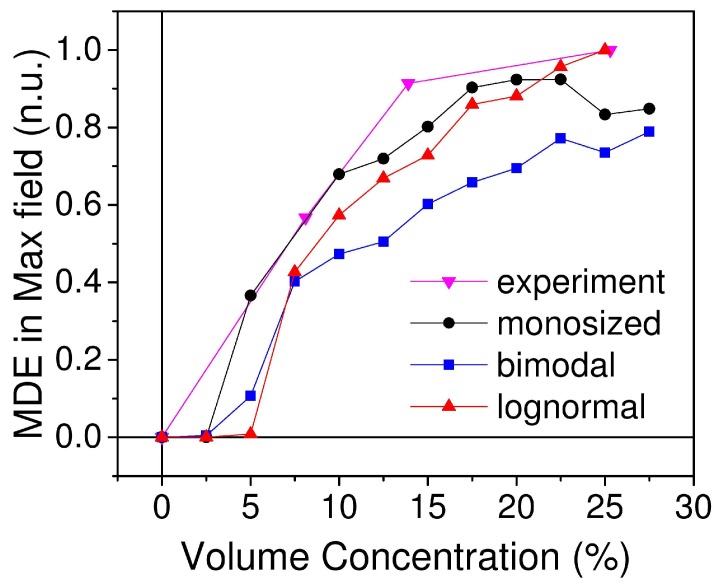
Volume concentration dependence of maximal MDE value for three types of size distribution compared to experimental data. Max field is equal to 955 kA/m.

MRE exhibit strong dependence of MDE on the magnetic particles mass/volume concentration. We performed series of simulations calculating the maximum value of MDE for varied value of the filler volume concentration for three types of particles size distribution. Simulations results were compared with experimental data obtained for samples described above (Figure 11). Mass fractions were recalculated to volume fraction. All three types of size distribution showed the same trend as experimental data. The lognormal type had the largest saturation value of MDE. For the small volume concentration, the lognormal distribution showed smaller MDE value in comparison with the other two types, and started to grow at higher concentration of magnetic filler. In the case of lognormal size distribution, there were fewer particles than for monosized sample due to the presence of larger particles. Since for low concentration the average distance between particles was large, the small number of particles played a significant role and led to the smaller value of effect.

**Figure 12:**
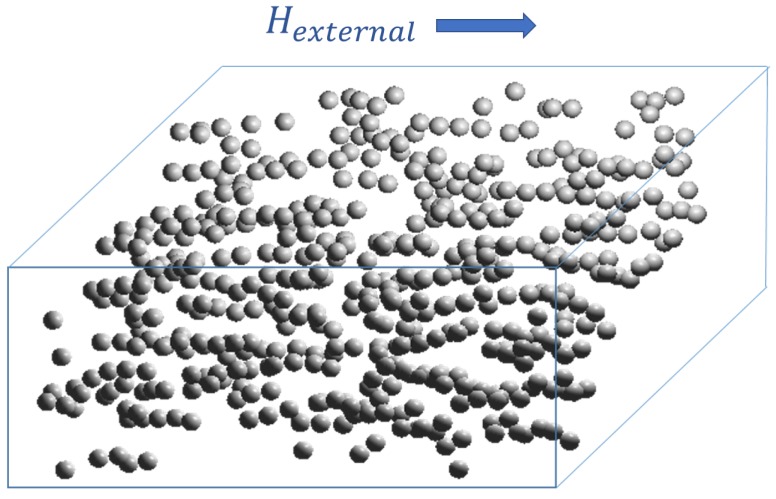
Formation of chain-like structures within the elastomer under applied magnetic field.

It is widely reported [31,32,33] that particles tend to form chain-like structures inside the MRE when external magnetic field is applied. We used GTK open library to visualize particles positions throughout the simulation. Visualization of aligned chains of particles inside the matrix in maximal magnetic field for monosized distribution is shown in Figure 12 and in Supplementary Videos.

### 3.4. Dependence of MDE on the Configuration of the Magnetorheological Elastomers (MRE) Sample

**Figure 13:**
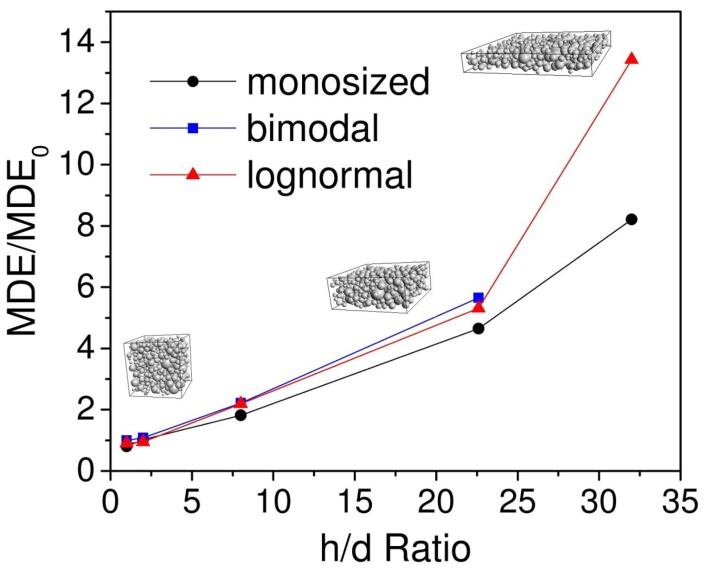
Increase of MDE value with increase of h/d ratio of the sample. h—side of capacitor (sample width); d—thickness of the sample (distance between the capacitor planes). $MDE_{0}$ is the value of effect for the sample of cubic shape (h/d = 1).

We performed a series of simulations to obtain the dependence of MDE on the sample shape. We changed width/thickness ratio of the MRE sample and calculated maximum MDE value for each case. Volume concentration of magnetic filler was fixed and equal to 27%. Volume of the sample was also fixed. The simulation showed the increase in the effect for the thinner samples. For lognormal type of size distribution, the increase was up to 1400% for ratio = 32 compared to ratio = 1 (Figure 13). This may be explained by the chains formed by particles being shorter, which led to increasing influence of the single particle displacement in the chain. Presence of big particles for lognormal type led to almost two times greater value of MDE than for monosized system. Significant increase of MDE value for thin MRE samples might be of high interest for applications in flexible electronics [34].

**Figure 14:**
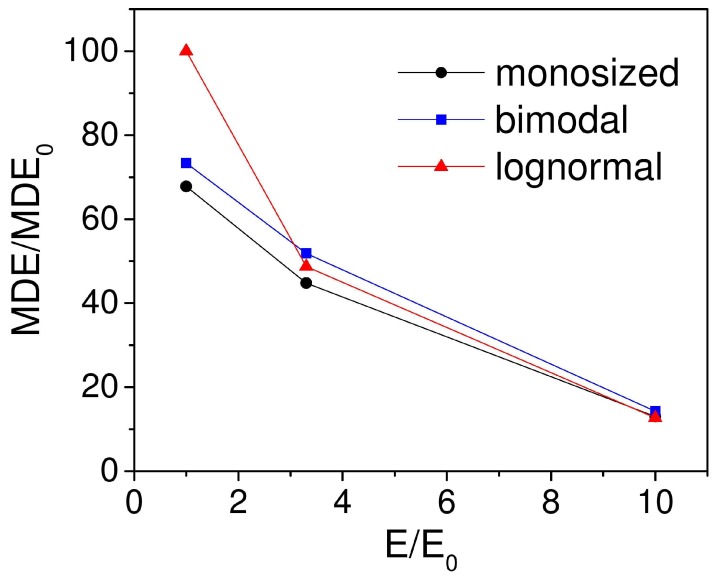
Decrease of MDE value with increase of Young’s modulus of the elastomer. $MDE_{0}$ is the value of MDE for average Young’s modulus $E_{0}$ = 0.01 GPa.

In addition, simulations showed the decrease of the effect magnitude with the increasing Young’s modulus of samples (Figure 14). All simulations were performed for cubic-shaped systems with volume concentration of 27%. Again, the lognormal distribution type showed the largest value for softer matrices, which was related to the presence of bigger particles in such distribution type. They created larger local magnetic fields and provided higher mobility, which led to larger MDE value among all three types of size distributions.

## 4. Experiment

Simulation data were compared with experimental results obtained by our group. Silicon compound SIEL^TM^ produced by the State Institute of Chemistry and Technology of Ogranoelement Compounds (Moscow) was used as soft polymer matrix. Carbonyl Iron particles with average diameter 5 μm were used as the ferromagnetic component. Synthesis of magnetorheological elastomers is described in a previous report [35].

In experimental part, samples with 40%, 54%, and 72% mass fractions of carbonyl iron were investigated. To compare with simulation results, volume fractions were used. Corresponding volume fractions were 8.1%, 13.9% and 25.3%. Magnetodielectric effect was calculated from the values of capacitance of capacitor filled with elastomer in the absence and in presence of constant magnetic field [15]. A sample of magnetorheological elastomer with length 18 mm, width 10 mm and height 3 mm was fixed between plates of capacitor and then placed between the poles of electromagnet so that the magnetic field was perpendicular to the capacitor plates. Before measurements, the sample remained for 5 min in the field for relaxation.

Capacitance was measured with RLC-meter AKTAKOM AM-3016 with frequency 50 kHz and amplitude 1 V at zero magnetic field and under applied field with flux density up to 900 mT. Field dependence of magnetization has been measured using VSM Lake Shore 7400 series with an average field sweep of 8 mT/s.

## 5. Conclusions

In this work, we present a model for simulating the magnetodielectric effect in magnetorheological elastomers. We performed a numerical study of the effect for series of samples with different concentration of magnetic filler, size and space distribution of particles as well as elastic properties of polymer matrix. The presented model provided good qualitative description of magnetic properties and magnetodielectric effect in magnetorheological elastomers. Good qualitative agreement with experimental data was achieved.

It was found that the type of particle size distribution significantly affected properties of the material. Lognormal type was shown to give better qualitative match of the modeling and experimental results than monosized type.

We found that the effect tended to saturation and had hysteretic behavior due to the elastic response of matrix. The influence of the orientation of magnetic field was studied as well, and the strong anisotropy of effect was observed.

## Supplementary Materials

Supplementary materials can be found at https://www.mdpi.com/1422-0067/20/6/1457/s1.

## Abbreviations

MRE	Magnetorheological elastomer
MDE	Magnetodielectric effect
VSM	Vibrating sample magnetometer
GTK	GIMP ToolKit
